# Transjugular Intrahepatic Portosystemic Shunt (TIPS) in Refractory Transudative Chylothorax due to Liver Cirrhosis

**DOI:** 10.1155/2020/2581040

**Published:** 2020-02-07

**Authors:** Muhammad Farhan Khaliq, Muhammad Muslim Noorani, Monica Chowdhry, Hesham Mohamed, Ashish Koirala

**Affiliations:** ^1^Department of Internal Medicine, Charleston Area Medical Center, Charleston, WV, USA; ^2^Department of Internal Medicine, Baptist South, UAB, Montgomery, AL, USA; ^3^Department of Pulmonary/Critical Care, West Virginia University, Charleston, WV, USA

## Abstract

Chylothorax is an infrequent type of pleural effusion, typically exudative, caused by obstruction or laceration of the thoracic duct by malignancy, trauma, or thoracic surgery. Transudative chylous pleural effusions are extremely rare. We report a case of a 63-year-old male with recurrent transudative chylothorax secondary to cirrhosis that completely resolved with transjugular intrahepatic portosystemic shunting (TIPS). Transudative chylous pleural effusion is an extremely rare entity with only a few cases reported in the literature to date. Transudative chylothorax can occur in patients with liver cirrhosis. Recognizing this association will prevent unnecessary testing and procedures. Timely diagnosis and early initiation of treatment are pivotal in preventing complications from malnutrition and infection by preventing loss of electrolytes, immunoglobulins, and T-lymphocytes.

## 1. Background

Chylothorax is a form of pleural effusion which results from the collection of chyle in the pleural space. A pleural fluid triglyceride level of ≥110 mg/dl is strongly suggestive of the presence of chylothorax [1]. Based on the etiologies, chylothorax can be divided into traumatic and nontraumatic causes [2]. The pathology lies in the lymphatic vessels, mostly the lymphatic duct. It is well known that patients with cirrhosis and portal hypertension have increased thoracic duct pressure and lymph flow [3–5]. It is thought that the pathophysiology of chylous ascites could be secondary to rupture of lymph vessels secondary to increased lymph flow [6], while chylothorax may be related to the migration of ascitic fluid from the diaphragmatic defect [7]. We present a case of transudative chylothorax in a cirrhotic patient who subsequently underwent TIPS placement.

## 2. Case Presentation

A 63-year-old Caucasian man presented to our emergency department with progressive shortness of breath that began 2 weeks prior. He denied any associated symptoms such as fever, weight loss, fatigue, chest pain, palpitations, lymphadenopathy, nausea, vomiting, or diarrhea. His medical history was significant for cirrhosis due to hepatitis C which was diagnosed 5 years ago. It was complicated with recurrent hydrothorax and refractory ascites. He failed a low-salt diet and maximal doses of diuretics. He required frequent admissions to other facilities every month for therapeutic thoracentesis and paracentesis for symptom relief. He achieved sustained virologic response for hepatitis C after treated with ledipasvir/sofosbuvir in the past. He denied any history of encephalopathy, hematemesis, or hematochezia. His other comorbidities included diabetes mellitus type II, chronic kidney disease stage III, and peripheral arterial disease.

In the emergency department, his vitals were normal. His physical exam included absent breath sounds on the right lower lobe. The abdomen was nontender but distended with shifting dullness. Cardiovascular and neurological examinations were unremarkable.

Initial laboratory studies obtained at our facility revealed total white cell count of 6.1 × 109, anemia with hemoglobin 9.2 g/dL, platelet count 52 × 109, blood urea nitrogen 43 mg/dL, creatinine 1.5 mg/dL, PT 12.3 seconds and INR 1.19. His liver function tests showed protein 8.1 g/dL, albumin 2.9 g/dL, alanine transaminase 133 unit/L, aspartate transaminase 189 unit/L, alkaline phosphatase 123 unit/L, total bilirubin 1.9 mg/dL, direct bilirubin 0.7 mg/dL, and LDH was 430 unit/L. His BNP was 103.

**Figure 1:**
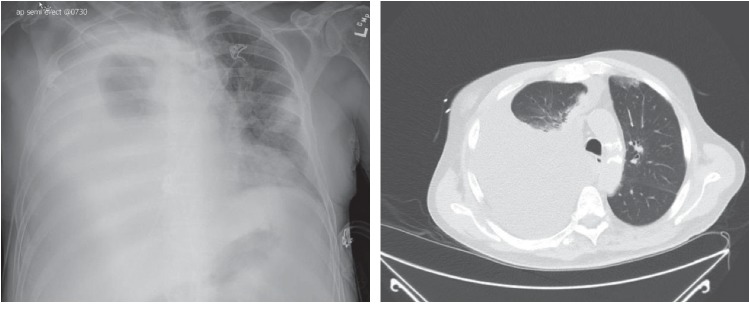
Chest X-ray and chest CT scan showing a right-sided pleural effusion.

Initial chest X-ray demonstrated a right pleural effusion. Computed tomography (CT) chest was subsequently performed that revealed large right-sided pleural effusion with the associated collapse of the right lower lobe (Figure 1).

Ultrasound of the liver confirmed changes secondary to cirrhosis and splenomegaly with a moderate amount of ascites; however, no focal lesions or masses concerning for malignancy were seen. CT abdomen was unremarkable with no signs of malignancy. Upper endoscopy did not reveal esophageal or gastric varices. Echocardiography did not demonstrate signs of heart failure or pulmonary hypertension.

The patient underwent thoracentesis and paracentesis. Thoracentesis revealed turbid yellow color fluid with pleural fluid differential which showed total nucleated cell count of 102 cells/mm^3^, red blood cell count of 19,301 cells/mm^3^, 41% neutrophils, 37% lymphocytes, and 22% other cells. Analysis of pleural fluid showed elevated triglycerides of 302 mg/dL consistent with chylothorax. His fluid pH was 7.73, albumin 0.6 g/dL, protein 1.1 g/dL, LDH 111 unit/L, glucose 152 mg/dL, and cholesterol 12. His pleural fluid protein/serum protein ratio was 0.13 and pleural fluid LDH/serum LDH ratio was 0.09, and serum-pleural fluid albumin gradient was 2.3 g/dl.

Paracentesis demonstrated cloudy red fluid with a pH of 8.0. Ascitic fluid differential showed total nucleated cell count of 168 cells/mm^3^, red blood cell count of 18,953 cells/mm^3^, 64% neutrophils, 17% lymphocytes, and 19% other cells. Analysis of ascitic fluid showed albumin level 0.2 g/dL, protein 0.5 g/dL, LDH 41 unit/L, glucose 145 mg/dL, and triglyceride 213 mg/dL. His serum-ascitic fluid albumin gradient was 2.7 g/dl.

## 3. Treatment

The patient was diagnosed with chylothorax after triglyceride levels were found to be greater than 110 mg/dL. He responded minimally to diuretics and sodium restriction. The patient refused parenteral nutrition. Based on his MELD-Na of 17 and poor responsiveness to medical management including diuretics and multiple therapeutic thoracenteses, the patient underwent transjugular intrahepatic portosystemic shunt (TIPS) procedure with a covered stent. His pre-TIPS portosystemic pressure gradient (PPG) was 17 mm Hg, and post-TIPS PPG was 8 mm Hg.

## 4. Outcome and Follow-Up

After undergoing the TIPS procedure, the patient was discharged home. No immediate complications from the procedure were noted. He required paracentesis and thoracentesis once. On 3-month, 6-month, and 1-year follow-up, patient repeat chest radiographs and abdomen ultrasound for recurrence of pleural effusion and ascites showed complete resolution of the fluid. He remained symptom-free without the need of further thoracentesis and paracentesis.

## 5. Discussion

Chylothorax is defined as pleural effusion characterized by a triglyceride level of greater than 110 mg/dl. It can be categorized into two classes based on traumatic and nontraumatic etiologies [2]. Traumatic etiologies could be iatrogenic or noniatrogenic. Iatrogenic traumatic causes include thoracic surgery including esophageal surgery, subclavian catheterization, and duct blockage due to central venous catheterization as a result of venous thrombosis [2]. Noniatrogenic traumatic causes that can lead to duct damage include fracture-dislocation of the spine, childbirth, and penetrating trauma from sharp objects. Nontraumatic etiologies are broadly classified into malignancy, idiopathic, and miscellaneous causes [2]. Lymphoma remains the most common nontraumatic cause of thoracic duct blockage [2]. If workup fails to establish etiology, it is termed idiopathic chylothorax [8, 9]. Other miscellaneous causes of chylothorax include sarcoidosis, hemangiomatosis, filariasis, amyloidosis, tuberculosis, cardiac failure, and cirrhosis [2]. Mostly, these chylous effusions are exudative; however, rarely transudative effusion has been reported in liver cirrhosis, nephrotic syndrome, carcinoid tumor, amyloidosis, lymphangioleiomyomatosis, SVC thrombosis, congestive heart failure, and constrictive pericarditis [10–14].

The etiology of chylothorax in cirrhosis is thought to be secondary to chylous ascites; however, isolated cases of chylous effusion due to cirrhosis have been reported in the absence of ascites [15]. Patients with cirrhosis and portal hypertension have elevated thoracic duct pressures. Because of this pressure and increased lymph flow (up to 20 liters/day), there is a rupture of serosal lymph vessels which contributes to chylous ascites [6]. The migration of chylous ascites into the pleural cavity is thought to be a secondary congenital defect in the diaphragm and possibly because of the negative intrathoracic pressures in the pleural cavity during inspiration [16].

The management of chylothorax related to cirrhosis includes intermittent thoracentesis for symptomatic relief of dyspnea. This fluid is rich in triglycerides, chyle, and lymphocytes, and frequent drainage could be cumbersome for the patient. It can lead to complications including malnutrition, volume depletion, and an immunocompromised state. A comprehensive management plan must include replenishment of volume and macro and micro (vitamins) nutrients. Usually, hyponatremia, acidosis, and hypocalcemia are noted in advanced cases [17].

For refractory cases of hepatic hydrothorax, TIPS has been performed with successful results. It works by decreasing postsinusoidal and portal pressure, thus decreasing hepatic and gastrointestinal lymph flow which constitutes most of the thoracic duct flow in patients with cirrhosis [16, 18, 19]. TIPS is also considered as a more effective treatment option for the control of ascites, and complete resolution of ascites is seen in up to 75% of patients [20]. Our patient remained symptom-free on 1-year follow-up.

In conclusion, chylothorax with cirrhosis is a rare complication of cirrhosis thought to be secondary to portal hypertension and elevation in thoracic duct pressure and flow rates. TIPS works by decreasing postsinusoidal and portal pressure with minor complications. We demonstrated that a patient with this condition can benefit from TIPS. More studies are needed to determine the use of indwelling pleural catheters as a bridge to TIPS or for palliative purpose in patients with recurrent pleural effusions secondary to cirrhosis.

## Abbreviations

TIPS: transjugular intrahepatic portosystemic shunt
CT: computed tomography
MELD: model for end-stage liver disease
TPN: total parenteral nutrition
PPG: portosystemic pressure gradient.

## Additional Points

*Learning Points*. (1)Transudative chylothorax is associated with liver cirrhosis. (2)Timely diagnosis and early initiation of treatment are pivotal in preventing complications. (3)Cases refractory to medical management should be treated with TIPS procedure.
